# Metastatic Malignant Melanoma Presenting as an Appendiceal Mucocele

**DOI:** 10.1155/2011/546570

**Published:** 2011-03-30

**Authors:** A. A. Alduaij, M. B. Resnick, M. Kawata, V. E. Pricolo

**Affiliations:** ^1^Department of Pathology, Rhode Island Hospital, The Warren Alpert Medical School at Brown University, Providence, RI 02908, USA; ^2^Division of Colon and Rectal Surgery, Robert Wood Johnson Medical School, Cooper University Hospital, University of Medicine and Dentistry of New Jersey, Camden, NJ 07103, USA; ^3^Division of Colorectal Surgery, Rhode Island Hospital, The Warren Alpert Medical School at Brown University, Providence, RI 02908, USA

## Abstract

Melanoma metastatic to the appendix is extremely rare. Here we describe a case of a 31-year-old female from Bolivia with a remote history of metastatic malignant melanoma first diagnosed as a cutaneous malignant melanoma ten years prior to this presentation. The patient was being followed for a mucocele which on resection was found to be metastatic melanoma. “Mucocele” is a generic diagnosis that warrants further characterization and treatment.

## 1. Introduction

Malignant melanoma involving the gastrointestinal (GI) tract is a rare condition although it is one of the most common malignancies having the potential to metastasize to the GI tract [1–4]. GI metastasis are frequently found during autopsy (50%–60% of cases), but only a small proportion of living melanoma patients are diagnosed with GI metastasis (2%–5% of patients) [5, 6]. The most common sites of melanoma metastasis to the GI tract are the stomach and small intestine [7, 8]. 

We report a case of malignant melanoma metastatic to the appendix presenting as acute appendicitis in the background of a mucocele. 

## 2. Case Presentation

**Figure 1:**
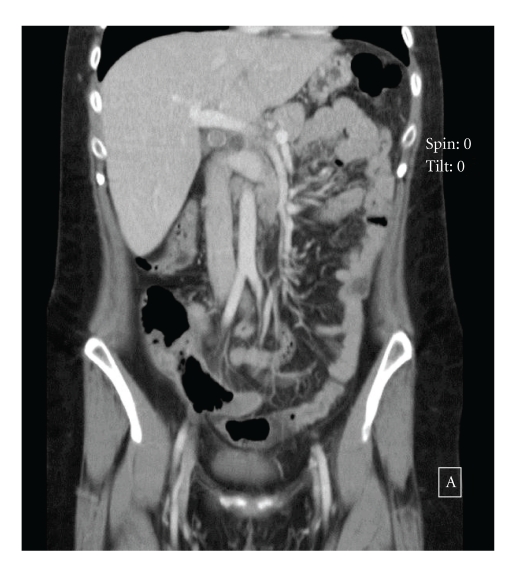
CT scan (coronal view) of the abdomen and pelvis showing an appendiceal mucocele with intraluminal gas and wall edema.

The patient is a 31-year-old female from Bolivia with a history of melanoma removed from her back ten years prior to this presentation. The pathology report was not available. In November 2005, the patient, now living in the USA, developed increasing headaches, vomiting, and blurred vision. CT scan showed a large left frontal cerebral mass with edema and displacement of the midline structures. The patient then underwent surgical resection of brain tumor which was interpreted as metastatic melanoma. After receiving palliative therapy, the patient enrolled in a chemotherapy trial, Temozolomide along with Sorafenib, at the University of Pennsylvania. Later in 2008, she developed a new 4 mm lesion in right frontal brain lobe. She had no evidence of other metastases. While the patient was being followed by serial CAT scans, a slow growing mass was noted in the appendix approximately one year prior to this presentation, which was interpreted as a mucocele and managed conservatively. In April of 2010, she presented to our institution with symptoms of acute appendicitis. A CT scan of the abdomen and pelvis revealed inflammatory changes and gas in the wall of the distended appendix (Figure 1). The mucocele extended to the base of the cecum; she underwent an appendectomy with partial cecectomy the same day to achieve adequate resection margins. 

Cloudy peritoneal fluid was noted intraoperatively, and the postoperative diagnosis was gangrenous appendicitis superimposed on a mucocele. She recovered uneventfully.

**Figure 2:**
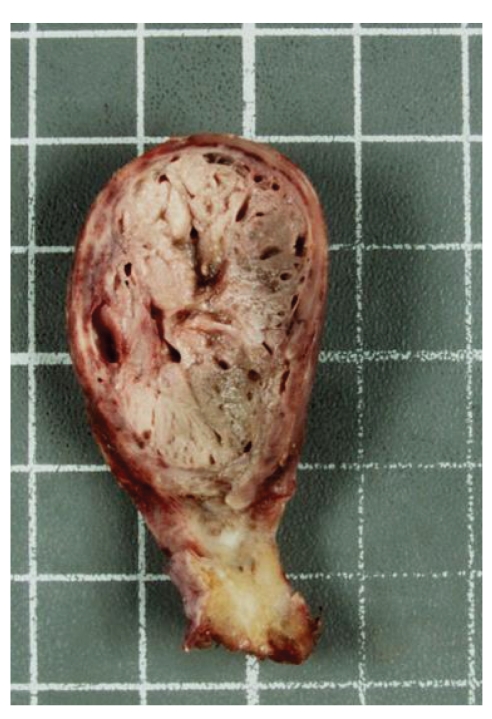
Gross examination: cross-section of appendix, lobulated green/brown partially hemorrhagic, and pigmented tumor occupy the proximal portion of the appendiceal lumen.

On gross examination, the appendix was 8.0 cm in length and 1.5 to 3.2 cm in diameter attached to a partial cecectomy. The serosal surface was smooth with areas of hyperemia, and a focal disruption measured 1.5 cm was located in mid appendix. Sectioning revealed an 7.5 cm in length with 1.4–3.0 cm in diameter tumor occupying more than 80% of the appendix. The tumor was soft, pink/tan in the distal aspect, and lobulated green/brown in the proximal portion (Figure 2). No perforation was grossly appreciated. 

**Figure 3:**
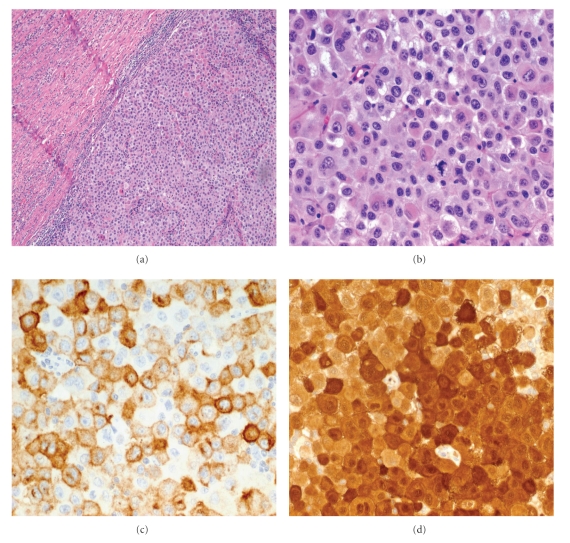
Microscopic examination: (a) solid cords and nests of tumor cells fill the appendiceal lumen and replace the appendiceal mucosa and submucosa with partial extension to the muscularis, no involvement of the serosa; (b) tumor cells with abundant eosinophilic cytoplasm, some of which are vacuolated, with pleomorphic nuclei, prominent nucleoli, and atypical mitosis (Hematoxylin and eosin staining, (a) x200; (b) x400). (c) Tumor cells strongly express HMB45, and (d) S-100 (HMB45 and S-100 immunostaining, x400).

On microscopic examination, tumor cells replaced most of the appendiceal mucosa and muscularis with no extension to the serosa. The lesional cells formed nests and cords and had an abundant eosinophilic cytoplasm with mildly pleomorphic nuclei and prominent nucleoli, some of which had intranuclear inclusions (Figures 3(a) and 3(b)). The tumor cells were positive for the melanoma markers Melanoma Antigen (M; HMB45; Enzo; New York), Melan-A (M; A103; Cell Marque; California), and S-100 (P; DAKO; California) (Figures 3(c) and 3(d)). Acute appendicitis and periappendicitis were also present. The cecum was free of disease. The diagnosis of metastatic malignant melanoma was made. The patient is doing well at the present time. She is alive with the disease. Plan for followup is to continue to monitor her metastatic melanoma and to enter patient in melanoma vaccine trials.

## 3. Discussion

Malignant melanoma represents 1–3% of all cancer in the USA [9]. Malignant melanomas develop from melanocytes which are derived from neural crest cells. The neural crest cells migrate during embryologic development and may be found in noncutaneous sites. Although they usually occur in the skin (cutaneous melanoma), melanomas can occur in any organ in which melanin-containing cells are present [7].

Melanocytes are normal residents of the mucous membranes of the upper aerodigestive tract, gastrointestinal, and urogenital tracts [7]. These cells give rise to malignant melanomas of the mucous membranes lining the GI tract. Malignant melanomas involving the GI tract may be primary (i.e., anorectum, esophagus, and gallbladder) or metastatic lesions (i.e., stomach and liver) [7]. Mucosal melanomas of the gastrointestinal tract are rare tumors that represent about 1.5%–2.0% of all melanomas [1, 7, 10]. The overwhelming majority of malignant melanomas involving the GI tract are secondary to metastatic disease [11]. The interval time between diagnosis of the primary and metastatic disease is variable (average, 7.0 years) [7]. Patients with GI metastasis may present with bleeding, anemia, obstruction, abdominal discomfort, pain, and intestinal perforation [7]. GI metastases usually appear as multiple polypoid lesions and can be either pigmented or amelanotic and often ulcerated [2]. Less commonly the presentation is of a solitary melanotic tumor. Metastases to the GI tract can present both at the time of primary diagnosis or years later as the first sign of recurrence. Diagnosis of metastatic melanoma is generally made by radiographic contrast studies, including CT, ultrasonography, PET scan and barium studies, and endoscopic evaluation. The sensitivity of CT for detecting metastases is only 60% to 70% [2, 12].

Metastatic melanoma has been observed in almost all regions of the human body. The most common sites of metastases were the lymph nodes (74%) and lungs (71%), followed by the liver (58%), brain (55%), bone (49%), adrenal glands (47%), and GI tract (44%), but only 1% to 4% of them are diagnosed antemortem [3]. Two large autopsy studies which looked at the distribution of metastases in the GI tract are from the Roswell Park Memorial Institute and Memorial Sloan Kettering Cancer Center. The distribution of GI organ metastases in both series was as follows: liver, 58–60%; peritoneum, 43%; pancreas, 38%; small bowel, 36–58%; spleen, 31%; colon, 22–28%; stomach, 20–23%; duodenum, 12%; rectum, 5%; esophagus, 4%; biliary tract, 9% [3, 5]. None of these studies or any others that we could find documented appendiceal involvement by metastatic melanoma.

We found one reported case describing a malignant melanoma in the vicinity of the appendix. The reported case was that of a 55-year-old Caucasian woman with no available medical history who presented with abdominal pain. Ultrasound suggested a periappendicular abscess or a tumor, but no other intraabdominal lesion was identified. The tumor cells in that case were positive for Melan-A, S-100, HMB45, and vimentin. No primary source of melanoma in this case was evident [13].

The prognosis of metastatic GI malignant melanoma is very poor with a 5-year survival of less than 10% [2, 14]. However, with surgical resection there may be a possibility of long-term, disease-free survival. Surgical resection of distance metastasis is still the mainstay of treatment. Studies have shown that surgical resection for melanoma metastatic to the GI tract may be effective for palliation and may also result in long-term survival in selected patients [4]. 

## 4. Conclusion

Preoperative diagnosis of appendiceal metastases from malignant melanoma is difficult but should be considered in any patient with a history of melanoma who develops GI symptoms, even in the absence of radiographic findings. Mucocele of the appendix should be managed with resection to ascertain its pathologic features and prevent pseudomyxoma peritonei or other complications.
